# Supplementary material for: Mental Health in Schoolchildren in Joint Physical Custody: A Longitudinal Study
Source: Children (Basel). 2021 Jun 4;8(6):473. doi: 10.3390/children8060473 (PMC8229623; doi:10.3390/children8060473)
Supplement: Supplementary file 1 [file children-08-00473-s001.zip › children-1248658-supplementary.pdf]

Supplemental material.

Table S1. Characteristics of the children living in a nuclear family at the six months' interview according to participation in the 11-year follow-up/inclusion in this study.

			Participant 11-year	Non-participant
			N=31,519	N=36,749
			%	%
Child gender	Boy		47.4	52.6
	Girl		52.8	48.4
Parental mental health				
	Psychiatric care: mother before birth of child		4.9	7.4
	Psychiatric care: father before birth of child		4.7	6.1
Maternal burden at 6 months				
	Mother burdened by relation to father			
	yes		12.5	12.7
	Mother burdened by economy			
	yes		18.3	19.7
Socio-demographic				
	Maternal age	16-22	1.9	3.5
		23-28	33.6	36.0
		29-34	48.5	46.0
		35+	15.9	14.5
	Maternal education at birth of child			
		Primary only	4.9	9.1
		Secondary	36.6	44.1
		1-3 post-secondary	43.0	35.6
		4 + post-secondary	15.5	11.2
	Paternal education at birth of child			
		Primary only	10.3	13.9
		Secondary	45.6	49.2
		1-3 post-secondary	27.3	23.8
		4 + post-secondary	16.8	13.0

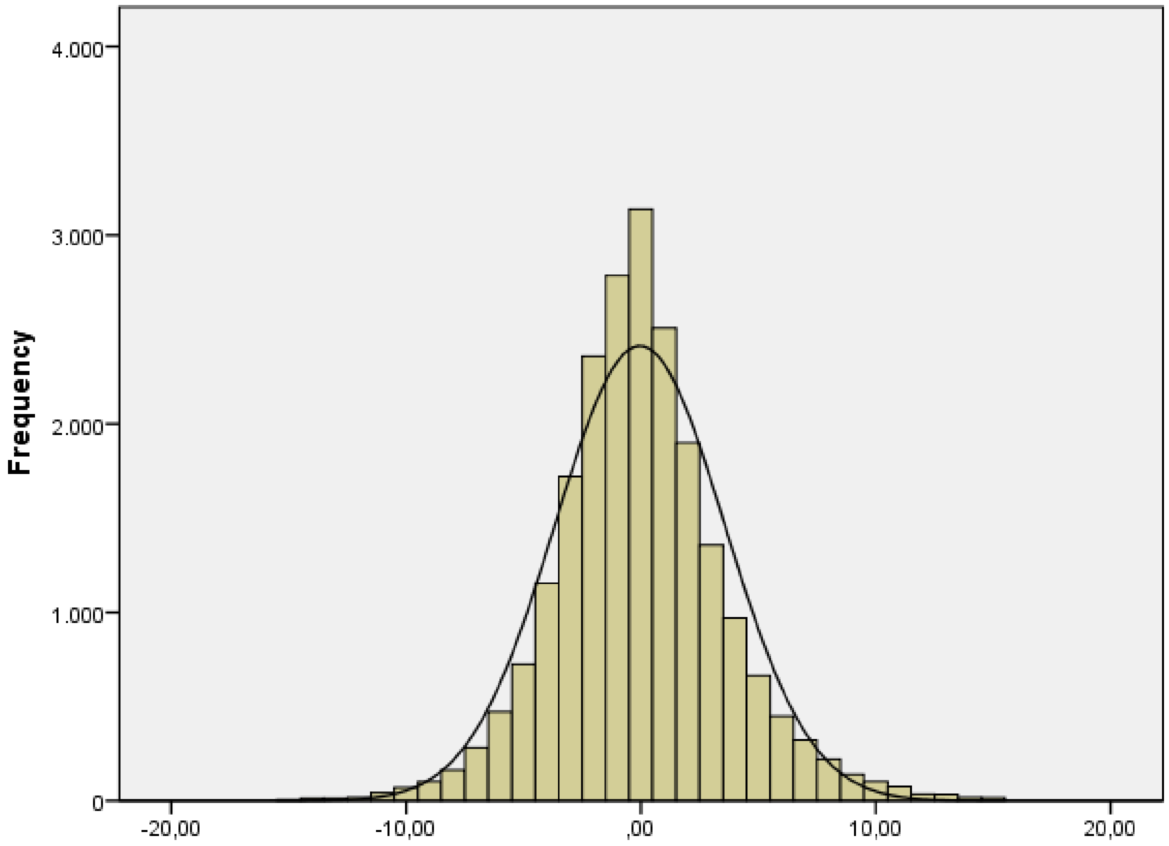

Figure S1. Histogram of distribution of change in total SDQ scores in maternal report from age 7 years to 11 years (N=24,479).
